# The Preparation and Potential Bioactivities of Modified Pectins: A Review

**DOI:** 10.3390/foods12051016

**Published:** 2023-02-27

**Authors:** Xu Jiao, Fei Li, Jing Zhao, Yunlu Wei, Luyao Zhang, Wenjun Yu, Quanhong Li

**Affiliations:** 1 College of Food Science and Nutritional Engineering, China Agricultural University, Beijing 100083, China; 2 National Engineering Research Center for Fruits and Vegetables Processing, Beijing 100083, China; 3 College of Life Science, Qingdao University, Qingdao 266071, China; 4 School of Life Science and Engineering, Southwest University of Science and Technology, Mianyang 621010, China

**Keywords:** pectins, modification, characterization, bioactivities

## Abstract

Pectins are complex polysaccharides that are widely found in plant cells and have a variety of bioactivities. However, the high molecular weights (M_w_) and complex structures of natural pectins mean that they are difficult for organisms to absorb and utilize, limiting their beneficial effects. The modification of pectins is considered to be an effective method for improving the structural characteristics and promoting the bioactivities of pectins, and even adding new bioactivities to natural pectins. This article reviews the modification methods, including chemical, physical, and enzymatic methods, for natural pectins from the perspective of their basic information, influencing factors, and product identification. Furthermore, the changes caused by modifications to the bioactivities of pectins are elucidated, including their anti-coagulant, anti-oxidant, anti-tumor, immunomodulatory, anti-inflammatory, hypoglycemic, and anti-bacterial activities and the ability to regulate the intestinal environment. Finally, suggestions and perspectives regarding the development of pectin modification are provided.

## 1. Introduction

Pectins are natural polysaccharides that are widely distributed in plants. According to their different structures, researchers divide pectins into homogalacturonan (HG), rhamnogalacturonan I (RG-I), rhamnogalacturonan II (RG-II), xylogalacturonan (XGA), and apiogalacturonan (AGA). The first three domains are relatively widespread, while the last two domains are found only in specific plants [1]. Natural pectins have been proven to have a variety of bioactivities. For example, natural citrus pectins prepared under alkali and high-pressure conditions exhibit potential prebiotic properties [2,3], natural acid-extraction okra pectin shows excellent anti-oxidant and anti-inflammatory activities [4,5], and natural hot-water-extraction pumpkin pectins possess immunomodulatory and hypoglycemic activities [6,7,8,9]. However, with the increasing depth of research, natural pectins are being reported to exhibit disadvantages in some specific areas. Li et al. (2021) suggested that the absorption in vivo is challenging for macromolecules [10], which means the high molecular weights (M_w_) and complex structures of natural pectins will limit their bioavailability. To overcome these problems, researchers focus more on the modification of pectin structures and properties.

The modification of pectins refers to the introduction of new functional groups into natural pectins or the implementation of changes to their M_w_ and molecular structures, which can significantly affect their properties. Researchers have found that modified pectins may exhibit preferable bioactivities compared to natural pectins. For example, Chaouch et al. (2018) found that the *Opuntia ficus indica* cladodes pectin possessed a new anti-coagulant activity after sulfation by sulfur trioxide-*N*,*N*-dimethylformamide (SO_3_-DMF) complex [11]. In addition, Huang et al. (2019) and Huang et al. (2020) suggested that *Mesona Chinensis* Benth and Chinese yam pectins sulfated by the chlorosulfonic acid-pyridine (CSA-Pyr) method showed stronger anti-oxidant and immunomodulatory activities compared to natural pectins, respectively [12,13]. Pectin modification can be classified into different methodologies, such as chemical, physical, and enzymatic methods. The chemical methods are the most traditional and widely used, mainly consisting of methyl esterification, acetylation, sulfation, and selenylation. In addition, they involve the modifications of acid, alkali, and oxidative degradation. Although chemical modification is used more frequently than other methods, the strict reaction process often requires the use of a large portion of organic solvent, which negatively impacts the environment [14,15]. The physical methods are a “green” and sustainable approach, using little or no harmful chemical reagents during the reaction. They mainly consist of ultrasound, high-pressure, subcritical, irradiation, and low-temperature plasma methods. Although physical modification is environmentally friendly, it is difficult to achieve selective modification using its random action mode [16,17,18]. Enzymatic modification is also known as a type of biological modification that can be applied to achieve targeted modification using enzymes corresponding to the pectin structures. Esterase, hydrolase, and lyase are commonly used as enzymes for this method. Enzymatic modification has the advantages of high specificity, safety, mild reaction conditions, and a lack of toxicity and side effects, and it can effectively avoid the substantial degradation of pectins during modification. Nevertheless, the expensive enzymes limit the widespread application of enzymatic modification in industrial production [19].

Based on the summary above, it is obvious that the modification methods and the potential effect of modification on bioactivities are essential points in developing modified pectin. With regard to the modification methods, chemical modification such as methyl esterification/de-methyl esterification, acetylation, sulfation, and selenylation have received considerable interest, while relatively little attention has been paid to acid, alkali, and oxidative degradation [13,20,21,22]. Physical modifications have been studied and described in depth in previous research [15], but there have been relatively few reviews on enzymatic modifications. Therefore, this article attempts to gather these diverse modification methods and review them from the perspective of basic points, influencing factors, and product identification. Furthermore, although some research has been done to assess the bioactivities of modified polysaccharides such as cellulose, dextran, and other neutral polymers [23,24], they have rarely focused on pectin, which is not conducive to the effective development of pectin modifications. Therefore, this article discusses the effects of modification on the bioactivities of pectin, including anti-coagulant, anti-oxidant, anti-tumor, immunomodulatory, anti-inflammatory, hypoglycemic, anti-bacterial activities, and the ability to regulate the intestinal environment. Finally, this paper also provides suggestions and perspectives on the development of pectin modification.

## 2. Chemical Modification of Pectins

Chemical modification is a widely used methodology for pectin modification since it can introduce new functional groups to pectin through chemical reagents. In addition, chemical modification can also change the M_w_, monosaccharide composition, molecular structure, and other macromolecular characteristics of natural pectins by acid, alkali, and oxidative degradation.

### 2.1. Introducing New Functional Groups

#### 2.1.1. Methyl Esterification

Methyl esterification is the most common method used to modify pectins. During the modification process, the free carboxyl groups (-COO^−^) contained in galacturonic acid (GalA) units are gradually esterified by methoxy, which causes pectins to convert from electronegativity to neutrality. In early research, pectins were first transformed into their tetrabutylammonium salt form and then dissolved in organic solvents, and the methyl esterification was completed by replacing -COO^−^ with dialkyl or dialkylaryl halides [25,26]. However, some researchers later discovered that the production of high-methoxyl pectins (HMP) by this method was accompanied by extensive depolymerization [27].

**Figure 1:**
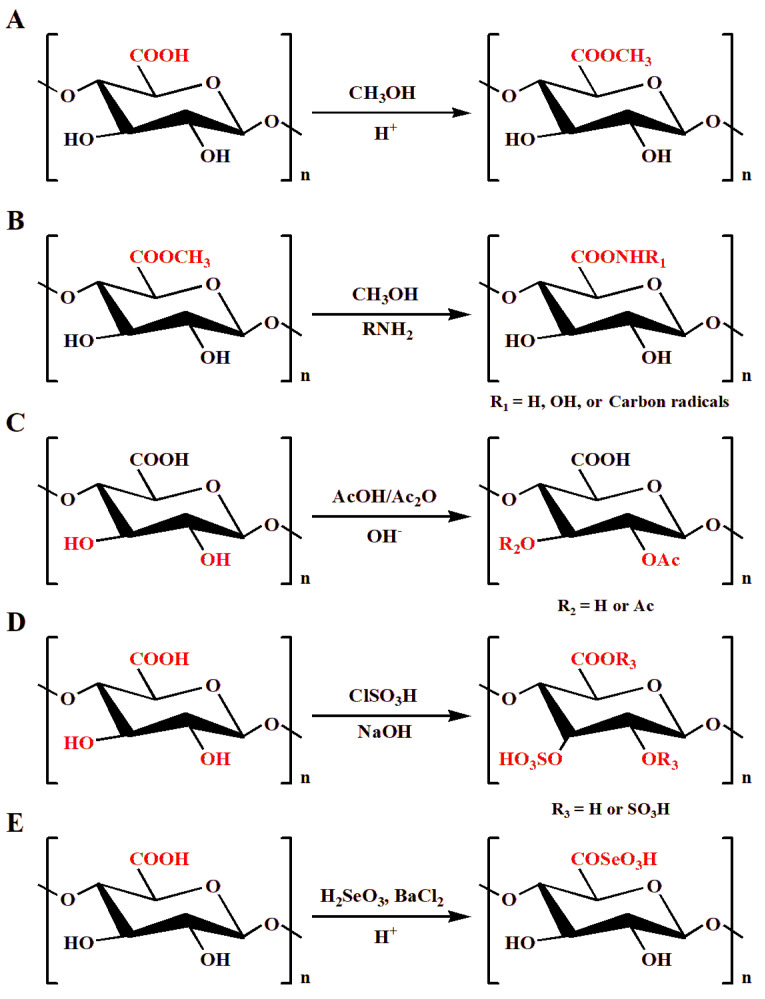
Methyl esterification (**A**), amidation (**B**), acetylation (**C**), sulfation (**D**), and selenylation (**E**) of pectin.

The acidified methanol method has been widely used for methyl esterification, in which pectins are directly methyl-esterified with methanol through the catalysis of acid [28]. As shown in Figure 1A, methyl esterification is a reversible condensation reaction between the -COO^−^ of pectins and the hydroxyl groups (-OH) of methanol. Therefore, reaction systems without water, such as anhydrous methanol, are more conducive to producing methyl-esterified GalA. The anhydrous methanol reaction system also takes advantage of a certain property of pectins, the fact that they are insoluble in methanol, to more easily and quickly separate the modified products after the reaction [29,30]. The acidified methanol method is influenced by the type and concentration of acid, as well as the reaction temperature and time. Generally, the higher and longer the reaction temperature and time are, the greater the degree of methyl esterification (DM) of pectins will be, but the stricter parameters of the process may also result in more severe degradation [30,31,32,33]. Sulfuric acid (H_2_SO_4_) and hydrochloric acid (HCl) are two catalysts commonly used in this method. However, when it comes to these acids, the reaction generally must be maintained at a low temperature (0–5 °C) to reduce the degradation of pectins, which means that a long reaction time (of perhaps several weeks) is required to prepare fully methyl-esterified products [20]. Addressing this drawback, Rosenbohm et al. (2003) reported that the acidification of anhydrous methanol with acetyl chloride instead of H_2_SO_4_ and HCl yielded modified pectins with a DM of 100% after 5 days at a reaction temperature of 5 °C [27]. Subsequently, Chen et al. (2022) also obtained modified pectins with a DM in the range of 90–100% by acidifying anhydrous methanol with acetyl chloride for 14 h at room temperature [34].

Methyl ester groups can not only be introduced into pectins but also be removed from them, which is called de-methyl esterification. The removal process is mainly completed by an alkali, acid, amidation reagent, and enzymatically, and their mechanisms are shown in Table 1. Alkali hydrolysis is relatively convenient and fast and is the most commonly used method for de-methyl esterification. However, the *β*-elimination reaction of polygalacturonic acid chains occurs simultaneously with de-methyl esterification, causing the degradation of pectins. Therefore, researchers generally perform the reaction at lower temperatures to reduce degradation [21,34,35]. Compared to alkali hydrolysis, acid hydrolysis results in great damage to the neutral sugar side chains of the RG-I domain, and the strict reaction conditions result in unstable yields and properties of the modified products, so it is not selected by most researchers. Modified low-methoxyl pectins (LMP) obtained by amidation treatment are called amidated low-methoxyl pectins (ALMP), but they will be limited by the toxicity of the amidation reagent [20,29]. Enzymatic treatment can remove the methyl ester groups without depolymerization, but the expensive enzymes render it difficult to industrialize this method [36,37]. The use of enzymes is described in detail in Section 4.1.1.

DM is used to evaluate the modification degree of pectins, which is defined as the ratio of methyl-esterified Gal*p*A to total Gal*p*A. According to the DM, pectins are divided into HMP (DM ≥ 50%) and LMP (DM < 50%) [34]. Titration is a widely used method for determining DM and is based on the formula “DM = V_2_/(V_1_ + V_2_) × 100%” (V_1_ and V_2_ are the volumes of alkaline solution for the first and second titration, respectively) [21,83,84]. In addition, Fourier transform infrared spectroscopy (FT-IR), nuclear magnetic resonance (NMR), gas chromatography (GC), high-performance liquid chromatography (HPLC), electrophoresis, and mass spectrometry can also be applied to determine DM [27,34,85,86].

#### 2.1.2. Acetylation

Acetylation uses acetylation reagents to introduce acetyl into the -OH present on the O-2 and O-3 of Gal*p*A in alkaline solutions. As shown in Table 1, there are three main factors affecting acetylation, namely the reaction solvents, acetylation reagents, and catalysts. The reaction solvent refers to the organic solvents used to disperse pectins, such as dimethyl sulfoxide (DMSO), formamide (FA), methanol, and dimethylacetamide (DMAc). Acetylation reagents include acetic acid (AcOH) and acetic anhydride (Ac_2_O), while catalysts include Pyr, methylimidazole, N-bromosuccinimide (NBS), and 4-dimethylaminopyridine (DMAP) [23,24,26]. Renear et al. (1999) tested the acetylation efficiency of DMSO-N-methylimidazole, DMSO-Pyr, and FA-Pyr systems and found that the FA-Pyr system could reach a higher degree of acetylation at the highest reaction rate [26]. In addition to the three abovementioned factors, acetylation is also generally proportional to the reaction temperature and time.

Similar to the DM, the degree of acetylation (DAc) is also used to evaluate the modification degree of pectins. The DAc is generally calculated by the formula “DAc = (1.62 × A%)/(43 − 0.42 × A%)” after the determination of the acetyl content (A%) by the hydroxylamine ferric chloride method [7,20].

#### 2.1.3. Sulfation

In recent years, sulfation has been increasingly applied to modify neutral polysaccharides rather than pectins. Sulfation uses sulfation reagents to replace the -OH of Gal*p*A with the sulfate groups. The commonly used sulfation reagents are CSA, H_2_SO_4_, sulfonylchloride, and sodium amine trisulfate. Organic solvents such as Pyr, FA, DMSO, trimethylamine, and toluene are usually used as media to relieve the harshness of these acidic reagents in regard to the pectins [20,32,38]. The degree of sulfation (DS) is generally proportional to the concentration of sulfation reagents and the reaction temperature and time. Although pectins modified by sulfation tend to exhibit lower M_w_ compared to natural pectins because of their degradation in the acidic conditions, excessively strict reaction conditions will result in severe degradation of the pectins, which is undesirable [31,32,33,39,40,41].

FT-IR and NMR spectra are often used to confirm whether the sulfation is successful. Generally, successfully sulfated pectins will show two new absorption peaks at around 810–830 cm^−1^ and 1230–1260 cm^−1^ in the FT-IR spectrum, which, respectively, represent the C-O-S and S=O bonds of the sulfate groups. In addition, the sulfation position of pectin can be determined according to the chemical shift of the signals in the NMR spectrum, which will generally be the O-2, O-3, and O-6 of Gal*p*A [33,40]. The DS is usually calculated by the formula “DS = (1.62 × S%)/(32 − 1.02 × S%) ” or “DS = (1.62 × S%)/(32 − 0.8 × S%) (the former regards -SO_3_Na as the substituent, while the latter regards -SO_3_H as the substituent) after the determination of the sulfate content (S%) by the barium chloride gelatin method [24].

#### 2.1.4. Selenylation

Since natural selenylated pectins are rare, artificial selenylated pectins have been drawing attention for the last eight years. As shown in Figure 1E, selenylation refers to a reaction in which the -OH of Gal*p*A is replaced with selenium functional groups by inorganic selenium, such as selenous acid, selenite, and selenium oxychloride in acidic media, including nitric acid and glacial acetic acid [22,42,43]. During selenization, inorganic selenium reacts preferentially with the hemiacetal hydroxyl (C6-OH), and this selenium mainly exists in the modified pectin in the form of selenium esters [87]. Selenylated pectins generally possess higher M_w_ than natural pectins because of the introduction of selenium. For example, Lee et al. (2017) and Tao et al. (2022) reported that the M_w_ of nature pectins increased by approximately 17–33% after selenylation [88,89]. Similar to other modification methods, selenylation is also proportional to the concentration of inorganic selenium, as well as reaction temperature and time before the modification achieves saturation [42].

FT-IR and NMR spectra are also used to confirm whether the selenylation is successful. Selenylated pectins should possess peaks located at around 600–700 cm^−1^ and 850–900 cm^−1^ in the FT-IR spectrum, which indicate the C-O-Se and Se=O bonds, respectively [42]. For the NMR spectrum, the 3.0–4.0 ppm signal intensities of selenylated pectins in the ^1^H-NMR spectrum may be weakened by changes in the proton chemical environment caused by the substitutions of selenium-containing groups [89]. In addition, selenylated pectins also tend to show a new peak at approximately 62–67 ppm in the ^13^C-NMR spectrum, which is related to the substitution of the O-6 position [43,88].

### 2.2. Degradation of Pectins

Due to the complex structures and high M_w_ of natural pectins, most of them cannot be degraded in the intestine, absorbed into the blood, or taken in by cells. It is well-known that the rate of cellular uptake of bioactive substances is closely related to their size. Generally, bioactive substances with smaller sizes or lower M_w_ are more easily taken in and thus better exhibit their bioactivities [10]. Therefore, lowering the M_w_ and simplifying the complex structures of pectins by degradation are conducive to the effective use of pectins in some application areas. In addition to the chemical degradation method mentioned in this section, the physical and enzymatic modifications mentioned in the following sections are also based on the degradation of pectins.

#### 2.2.1. Acid Degradation

Acid treatment depolymerizes pectins by using differences in tolerance between different glycosidic bonds to acids. As the mechanism shown in Table 1, the neutral sugar side chains of pectins are attacked first and degraded into mono- or oligosaccharides, and then the backbones are attacked in acidic environments. However, polygalacturonic acid composed of α-(1→4)-D-GalA has excellent tolerance to acid and can hardly be cleaved [44,45,46].

The degree of acid degradation is proportional to the reaction temperature and time. In addition, it is also influenced by acid type and concentration. HCl, H_2_SO_4_, and trifluoroacetic acid (TFA) are commonly used to degrade pectin. Garna et al. (2006) degraded pectins using the three abovementioned acids and found that TFA caused less damage to polygalacturonic acid than HCl and H_2_SO_4_ [47]. In fact, TFA is often used to remove the neutral sugar side chains of pectins, especially those composed of arabinose (Ara). Li et al. (2018) and Zhang et al. (2012) continuously degraded RG-I-type pectin with 0.1 M TFA, and the results showed that with the increasing reaction time, Ara could be completely removed, while galactose (Gal) was almost unaffected by the acid environment [48,49]. The acid degradation of pectins has the advantages of high reaction rates (especially under heated conditions) and complete reaction degrees, but its reaction conditions are severe and the products have high monosaccharide contents, which is unfavorable for their subsequent study.

#### 2.2.2. Alkali Degradation

Alkali treatment depolymerizes pectins by taking advantage of the *β*-elimination reaction of polygalacturonic acid in alkaline environments, and the related mechanism is shown in Table 1. The *β*-elimination is more likely to occur on the glycosidic bonds between the esterified Gal*p*A. Therefore, HMP is more subject to *β*-elimination than LMP [90].

Similar to acid degradation, the degree of *β*-elimination reaction is also proportional to the reaction temperature and time. Zhang et al. (2021) degraded pectin with alkali solutions at 25 °C and 3 °C, respectively, and the results showed that the former could obtain modified products with lower M_w_ and GalA contents [51]. The *β*-elimination reaction is also influenced by the pH values and cations of the reaction systems. Kirtchev et al. (1989) suggested that not only *β*-elimination but also the demethylation reaction, which is mutually inhibited in conjunction with it, will occur in alkaline environments [52]. Specifically, *β*-elimination reaction will be dominant in reaction systems with lower pH values; otherwise, de-methyl esterification will be dominant. Regarding cations, Sajjaanantakul et al. (1993) found that the increase in the cation concentration could promote the *β*-elimination reaction, and the promoting effect of divalent cations is stronger than that of monovalent cations [53].

#### 2.2.3. Oxidative Degradation

The oxidative degradation of pectin is based on a Fenton reaction. Oxidizing groups, such as hydroxyl radicals (·OH) and superoxide anion radicals (·O_2_^−^), can combine with the hydrogen atoms attached to the carbon atoms and then attack the glycosidic bonds [54].

The metal Fenton reaction takes advantage of metals or their oxides, especially Fe^2+^, as catalysts to catalyze the generation of oxidizing radicals from H_2_O_2_. Zhi et al. (2017) and Yeung et al. (2021) degraded pectin with 96–98% M_w_ loss using the Fe^2+^-H_2_O_2_ system over 1–2 h, and the latter authors believed that these degradations occurred mainly in the HG domain and the neutral sugar side chains of the RG-I domain [55,56]. The degree of the metal Fenton reaction is generally proportional to the reaction temperature and time, as well as the Fe^2+^ and H_2_O_2_ concentrations when the reaction has not reached saturation. Although these metals and their oxides were discovered at an early stage of research and have been used as catalysts for Fenton oxidation systems, they are difficult to separate after the reaction, which is unfavorable for polymers that need to be used in food systems.

The non-metal Fenton reaction takes advantage of physical techniques such as ultraviolet (UV) light, ultrasound (US), and microwaves, instead of metal, as catalysts to generate oxidizing radicals without any separation process after the reaction. Cao et al. (2020), Hu et al. (2019), and Li et al. (2018) degraded pectins by UV light and US-assisted H_2_O_2_ systems and finally obtained pectins rich in the RG-I domain [57,58,59]. In addition to the influence of the H_2_O_2_ concentration, reaction temperature, and time, the non-metal Fenton reaction is also affected by the physical parameters of the process, such as the ultrasonic intensity and microwave power. Notably, a higher ultrasonic intensity does lead to a better degradation effect, but an excessive ultrasonic intensity may generate more cavitation bubbles, which pose obstacles to energy transfer, and result in a decrease in the degradation efficiency [58].

Based on the above analyses, it is apparent that the glycosidic bonds between different monosaccharides exhibit different levels of sensitivity to the environment. As described in Section 2.2.1, the glycosidic bonds between neutral sugars are most sensitive in acidic environments, while the glycosidic bonds between Gal*p*A are highly tolerant toward acids. However, in oxidative degradation, the HG domain is preferentially attacked by oxidizing radicals, and the bonds between neutral sugars are almost unaffected in the initial degradation stage. Therefore, oxidative degradation is often used to prepare pectins rich in the RG-I domain [55,57,58,59].

## 3. Physical Modification of Pectins

Although chemical modification is widely used because of its low cost and wide availability, it requires high-quality anti-corrosion equipment and may cause serious environmental pollution. In contrast, physical modification requires little or no chemical reagents, which can avoid additional purification processes, and has the advantages of simplicity, speed, and economy. However, due to mechanical limitations, physical modification is difficult to implement in industrial production and it cannot degrade pectins selectively [14,15,31]. Physical modification mainly consists of ultrasound, high-pressure, subcritical water, and irradiation modifications.

### 3.1. Ultrasound Modification

Ultrasound (US) treatment depolymerizes pectins by using the chemical and physical (or mechanical) effects induced by ultrasonic cavitation. Compared with other physical modification methods, ultrasound treatment can better control the degree of pectin depolymerization and has a shorter processing time. In the initial stage of ultrasonic power, cavitation bubbles are formed, resulting in compression and expansion. As the cavitation bubbles implode, high shear forces are generated, which can break the glycosidic bonds in the pectins, a process that is also known as a physical (or mechanical) effect. In addition, the collapse of the cavitation bubbles promotes the dissociation of water molecules to produce -OH and -H radicals and the formation of H_2_O_2_ to attack the glycosidic bonds, which is a chemical effect [15,54,60,61]. Notably, ultrasound modification is more effective for the side chains of pectins rather than the backbones because the latter is more resistant to US [91].

Ultrasound modification is mainly affected by the US intensity/frequency, duty cycle, reaction temperature, time, and pH value. The degree of ultrasound modification is proportional to the first four factors in most cases [16,58,62,63,64]. As for the reaction pH value, Yan et al. (2020) suggested that as the pH value of the reaction system increased (4.0 to 10.0), some of the Gal*p*A in pectins were converted into -COO^−^, which enhanced the electrostatic repulsion between the pectin molecules and transformed them into more easily attackable “stretch” structures [91].

Although ultrasound modification is considered to be one of the most effective “green” degradation techniques, the attenuation of energy transmission under prolonged or high-intensity ultrasonic fields limits the degradation of pectins by US. Therefore, researchers combine ultrasound with other modification methods to improve the degradation degree of pectin, such as US-assisted oxidative (described in Section 2.2.3), high-pressure, and enzymatic modifications. For example, Larsen et al. (2021) modified pectin by the US-assisted enzymatic method. They found that, on the one hand, US could depolymerize pectins into oligomers with medium M_w_ and fewer branches, which were more easily attacked by enzymes. On the other hand, it improved the enzyme activities of polygalacturonase and pectin lyase [92]. In addition, Ma et al. (2016) combined US and enzyme to modify pectin, and the results suggested that, compared with the degradation with enzyme only, the addition of ultrasound could significantly reduce the DM but retain the DAc of pectin [93].

### 3.2. High-Pressure Modification

High-pressure modification mainly consists of high hydrostatic pressure (HHP) and high-pressure homogenization (HPH) [15]. As shown in Table 1, HHP and HPH depolymerize pectins by pressure transferred through the liquid medium and the forces generated by fluid flowing through a small gap in a short period of time [65]. Furthermore, there is another new processing method based on HPH, namely dynamic high-pressure microfluidization (DHPM), which combines conveying, pressurizing, mixing, and ultra-micro-crushing actions and can generate a huge pressure to change the structure of polymers in a short time [94,95].

High-pressure treatment mainly acts on the methyl ester groups (related to the DM) and side chains of pectins. There are some controversies related to the changes in the DM during high-pressure modification. Some research has suggested that high-pressure treatment cannot significantly influence the DM of pectin [66,96], while others believe that the mechanical force generated by the high pressure could cleave the C-O bonds of the -COO^−^ and lower the DM [97]. For example, Arachchige et al. (2020), Sun et al. (2019), and Xie et al. (2018) lowered the DM of pectins by approximately 19–50% through high-pressure modification [65,98,99]. In addition, some research results showed slight DM increases in pectins after high-pressure treatment. Zhong et al. (2021) and Zhang et al. (2017) obtained pectins with a DM increase of approximately 5–26% after modification [67,100]. A possible explanation for these controversies is that differences in the sources and structures of pectins can affect the results of high-pressure modification. The pectin side chains appear to be more easily cleaved by high pressure than the backbones [67,101,102]. Xie et al. (2018) found no significant changes in the GalA content and Rha/GalA ratio of pectin after high-pressure treatment, which represented the retention of pectin backbones. However, they also noticed a decrease in the ratio of (Ara + Gal)/Rha, which reflected the degradation of the pectin side chains [65]. Similar results were also observed in the research of Zhong et al. (2021) [67].

It is worth noting that since the high-pressure process can stabilize or even activate some enzymes, it is also often used to assist enzymes in degrading pectin [103]. Ma et al. (2013) used HHP-assisted endo-polygalacturonase to prepare pectin oligosaccharides, and the results showed that the oligosaccharide yields of the combined process at 300 MPa were significantly higher than those of the conventional enzymatic method [104]. Wan et al. (2019) used HHP-assisted pectin methylesterase to perform de-methyl esterification and found that the combined process required only one-tenth of the time required using the conventional enzymatic method to obtain a similar effect [103].

High-pressure modification is mostly affected by process pressure. Generally, the higher the pressure is, the greater the degradation of pectin will be [66], but there are also studies that have arrived at the contrary conclusion. Zhong et al. (2021) found that the M_w_ of pectins increased with increasing pressure when pectin was degraded by high-pressure modification in the range of 0.1–400 MPa, and it did not decrease until the pressure increased to 600 MPa [67]. In addition to the process pressure, the high-pressure modification may also be affected by the solution pH value, temperature, and cycle number (for HPH and DHPM), but these factors have much less influence on the modification than the process pressure.

### 3.3. Subcritical Water Modification

Water above boiling but below critical point (100–374 °C) is called subcritical water, which depolymerizes pectins by hydrolysis. Klinchongkon et al. (2017) lowered 97% of the M_w_ of pectin using subcritical water at 160 °C and 5 MPa in 5 min [71]. Pińkowska et al. (2019) degraded pectins using 155 °C and 36 min subcritical water treatment to prepare a large number of uronic acids [105]. Although the degradation of pectins by subcritical water is non-selectable, some organic acids such as malic acid, oxalic acid, and citric acid are added to the reaction system to induce selective catalysis. Ramirez et al. (2021) added organic acids to a subcritical water system, and the results suggested that the side chains of pectin were degraded first, followed by the backbones. They also found that there were different catalytic effects between the organic acids. Specifically, malic acid was more conducive to releasing Ara, xylose (Xyl), glucose (Glc), and Rha, while citric acid favored the release of Xyl, Ara, and fucose (Fuc) [69,106].

Since subcritical water cleaves the glycosidic bonds in pectins—mostly by the thermal effect—the reaction temperature is the main factor influencing subcritical water modification. Generally, the higher the reaction temperature, the greater the degradation of pectin will be. Ramirez et al. (2021) found that compared to subcritical water at 125 °C, the degradation of pectin with subcritical water at 135 °C could significantly produce more oligogalacturonides with a degree of polymerization (DP) of 2–3 [69]. Klinchongkon et al. (2017) compared the degradation of pectins by subcritical water at different temperatures and showed that the higher the reaction temperature is, the lower the M_w_ of the products will be [71,72]. It is worth noting that excessive temperature is not conducive to modifying pectins because it can promote the generation of advanced glycation end-products, which are undesirable [70]. In addition to temperature, the reaction pressure and time also influence subcritical water modification, albeit to far lesser extent than the reaction temperature [69,71].

### 3.4. Irradiation Modification

Irradiation modification induces physical and chemical changes in pectins by gamma irradiation or electron beams. The former has a longer treatment time but deeper penetration depth, while the latter requires a shorter residence time but can only penetrate a few centimeters below the sample surface [17,73,74]. Kang et al. (2006) prepared pectin oligosaccharides with a M_w_ less than 10 kDa using Co-60 gamma rays, and Gamonpilas et al. (2021) obtained modified pectin with 98–99% M_w_ losses by electron beam irradiation [17,107].

Irradiation modification mainly acts on the M_w_ and DM of pectins and is mostly influenced by the irradiation dosage. For M_w_, the higher the irradiation dosage is, the lower the M_w_ will be, and this degradation effect may be more apparent at low irradiation dosages. There are still some controversies regarding the DM. Gamonpilas et al. (2021) found that the DM of pectin increased with the increasing irradiation dose when the dose was higher than 50 kGy, but this phenomenon was only observed when the DM was detected by FT-IR. When the DM was detected by HPLC, there was no significant change in 125 kGy [17]. However, Ayyad et al. (1990) found that Co-60 gamma rays could lower the DM of pectins by approximately 8% (the DM was determined by HPLC) [75]. Sjöberg et al. (1987) treated whole apples with Co-60 gamma rays and showed that the DM of the pectin isolated from the irradiated apples was lowered by approximately half compared to that of the pectin isolated from the untreated apples (the DM was determined by gas–liquid chromatography) [76]. Some possible explanations for these discrepancies are the differences in the accuracy of detection means.

### 3.5. Low-Temperature Plasma Modification

Plasma is a mixture of positive ions and electrons produced by applying energy to a gas or gas mixture through ionizing gas, and it contains low-temperature and thermal plasma. Low-temperature plasma has been used to degrade pectins, and the detailed mechanism is shown in Table 1. Momeni et al. (2018) and Basak et al. (2022) lowered the M_w_ and DM of pectin by 18% and 11% and by 21% and 75% through nitrogen glow discharge low-temperature plasma and atmospheric pressure pin-to-plate cold plasma, respectively [80,81,108].

Low-temperature plasma modification is mainly influenced by the treatment voltage and time. Generally, the higher and longer the treatment voltage and time are, the more reactive species can be generated, which degrade the pectins to a greater extent [80,81,82].

### 3.6. Other Physical Modifications

In addition to the physical modifications mentioned above, some uncommon methods have been used to modify pectins. For example, Calce et al. (2012) used microwave instead of traditional heating to modify pectin by acylating the alcoholic functions of the polysaccharide using several fatty acid anhydrides in a short time (3–6 min) [109]. Ma et al. (2013) used the pulse-assisted modification of pectin with arachidic anhydride and prepared pectin arachates with a DM of 49–84% [110]. Chen et al. (2014) lowered the M_w_ of pectin by a maximum of 92% through high-speed shearing [111].

## 4. Enzymatic Modification of Pectins

**Figure 2:**
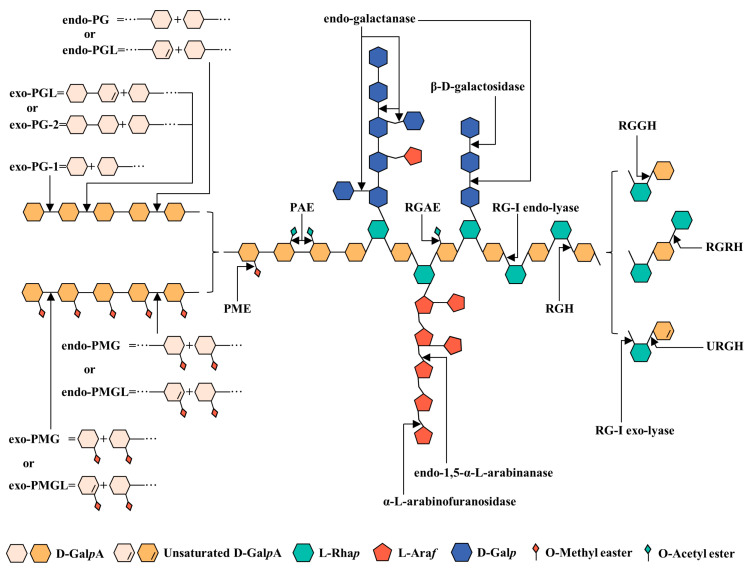
A summary of the action modes of enzymes used to modify pectin.

Compared with chemical and physical modification, the greatest advantage of enzymatic modification is the high specificity. In addition, it has the benefits of mild reaction conditions, a high degradation efficiency, and being friendly to the environment. However, the high specificity also means that the modification requires the cooperation of multiple enzymes to achieve the desired degradation effect, which results in high costs [19]. According to their different specificities, enzymatic modification can be divided into modifications of the backbones and side chains as shown in Figure 2. The sites and production of enzymes are listed in Table 2.

### 4.1. Modification of Pectin Backbones

Pectin backbones include polygalacturonic acid and rhamnogalacturonan. The former consists of the repeating →4)-α-Gal*p*A-(1→ units that exist in the HG, RG-II, XGA, and AGA domains and is sensitive to pectin esterase, hydrolase, and lyase. The latter consists of the repeating →4)-α-Gal*p*A-(1→2)-α-Rha*p*-(1→4)-α-Gal*p*A-(1→ units that exist in the RG-I domain and is sensitive to RG-I acetylesterase, hydrolase, and lyase [128].

#### 4.1.1. Pectin Esterase

Pectinesterase consists of pectin methylesterase (PME) and pectin acetylesterase (PAE), which remove the methyl and acetyl groups from polygalacturonic acid to produce methanol and ethanol, respectively. Currently, there is more research on PME than PAE. For example, Zhang et al. (2022) and Zhou et al. (2021) used PME to lower the DM of pectins by 52% and 90%, respectively [36,37]. Pillai et al. (2020) also used PME to de-methyl-esterify natural high-methoxy pectin and obtained a series of modified pectins with a DM distribution of 33–42% [129]. Notably, PME produces highly toxic methanol while de-methyl esterifying, which is unfavorable for pectins that are modified and subsequently used in food systems.

#### 4.1.2. Pectin Hydrolase

Pectin hydrolase depolymerizes pectins by attacking the α-1,4-glycosidic bonds between Gal*p*A units to generate pectin oligosaccharides or mono-Gal*p*A. According to their different substrates, pectin hydrolases can be divided into polygalacturonase (PG) and polymethylgalacturonate (PMG), which attack the α-1,4-glycosidic bonds between unesterified and esterified Gal*p*A units, respectively. Moreover, PG and PMG can be further divided into endo-PG and exo-PG, in the former case, and endo-PMG and exo-PMG, in the latter case, according to their mechanisms of action. The endo-fashion enzymes hydrolyze polygalacturonic acid chains in a random fashion, releasing shortened pectin oligosaccharides, while the exo-fashion enzymes hydrolyze the chains from the terminal end, releasing mono-Gal*p*A or disaccharides [114]. Although pectin hydrolases contain many subsidiary enzymes, endo-PG is the most common and widely used enzyme in current research. Humerez-Flores et al. (2022) used endo-PG to hydrolyze pectin rich in the HG domain and finally obtained pectin oligosaccharides with an M_w_ loss of 83–89% and DP of 1–5 [130,131]. Oosterveld et al. (2002) and Combo et al. (2013) combined endo-PG with PME to prepare a variety of modified products with different GalA contents [132,133].

#### 4.1.3. Pectate Lyase

Pectate lyase also depolymerizes pectins by attacking the α-1,4-glycosidic bonds between Gal*p*A units, but unlike pectate hydrolase, it cleaves the glycosidic bonds at the C-4 positions of Gal*p*A through the *β*-elimination reaction and eliminates H atoms from the C-5 positions, resulting in the generation of unsaturates in a process that is the same as the mechanism of alkali degradation. According to the substrate and mechanism, pectin lyases can also be divided into polygalacturonate lyase (PGL) and polymethylgalacturonate lyase (PMGL) in endo- and exo-fashion [60,121,134,135,136]. The research on PGLs is more prevalent. For example, Sukhumsiirchart et al. (2009), Zheng et al. (2013), and Zheng et al. (2018) degraded pectins into unsaturated di- and trigalacturonic acids and oligosaccharides with a DP of 1–6 using immobilized, high-, and low-temperature-resistant PGLs [137,138,139].

#### 4.1.4. RG-I Acetylesterase, Hydrolase, and Lyase

Similar to the degradation of polygalacturonic acid, enzymes that degrade RG-I backbones can also be divided into acetylesterase, endo/exo-hydrolase, and endo/exo-lyase.

RG-I acetylesterase (RGAE) only hydrolyzes acetyl groups on the O-2 or O-3 of GalAs in RG-I backbones. Although RGAE acts in a similar manner to PAE, they are suitable for different substrates [122].

RG-I endo-hydrolase (RGH) attacks the α-1,2-glycosidic bonds between the Gal*p*A and Rha*p* of RG-I backbones, releasing oligosaccharides with Gal*p*A and Rha*p* at the reducing and non-reducing end, respectively. RG-I exo-hydrolase contains RG-I galacturonohydrolases (RGGH), RG-I rhamnohydrolases (RGRH), and unsaturated rhamnogalacturonyl hydrolases (URGH). RGGH and RGRH cleave the α-1,2- and α-1,4-glycosidic bonds between Gal*p*A and Rha*p* in the non-reducing terminus of RG-I backbones, releasing Gal*p*A and Rha*p*. In contrast, URGH only degrades RG-I oligomers with unsaturated Gal*p*A at the non-reducing end, cleaving the α-1,2-glycosidic bonds between the unsaturated Gal*p*A and Rha*p* and releasing unsaturated Gal*p*A [122,140,141].

RG-I endo-lyase cleaves the α-1,4-glycosidic bonds between the Gal*p*A and Rha*p* of RG-I backbones (*β*-elimination), releasing oligosaccharides with Rha*p* and unsaturated Gal*p*A at the reducing and non-reducing end, respectively. RG-I exo-lyase cleaves the α-1,4-glycosidic bonds of RG-I oligomers with Rha*p* at the reducing end and unsaturated Gal*p*A at the non-reducing end, releasing disaccharide and reducing the size of rhamnogalacturonan with unsaturated Gal*p*A at the non-reducing end [122,142,143].

In the case of these abovementioned enzymes acting on the pectin backbones, the long and complex side chains often prevent them from reaching the action site smoothly, resulting in a low degradation efficiency. Therefore, researchers usually first remove these side chains using acids (described in Section 2.2.1) or enzymes aimed toward the side chains, enabling the more complete degradation of the pectin.

### 4.2. Modification of Pectin Neutral Side Chains

Although both the RG-I and RG-II domains have branches, more research has focused on the neutral sugar side chains of the RG-I domain. The RG-II domain has the most complex neutral sugar side chains in pectins, containing 11–12 glycosides, 28–36 sugars, and more than 20 glycosidic bonds [144], and it is not easily degraded by enzymes. As for the RG-I domain, there are three typical neutral sugar side chains, namely galactan (β-1,4-linked galactose), arabinan (α-1,5-linked arabinose with some Araf substitutions at C-2 or C-3), and arabinogalactan (AG). AG is further divided into type I (AG-I) and type II (AG-II) structures. The former is composed of 1,4-linked Gal units with an Ara or Galp substituent on the O-3 or O-6 of Gal, while the latter is composed of 1,3,6-linked Gal units with 1,3- and 1,6-galactan chains covered with Araf [48,140,145].

For the neutral sugar side chains mentioned above, endo-arabinanase, α-L-arabinofuranosidase, endo-galactanase, and β-D-galactosidase cleaved the glycosidic bonds in endo- and exo-fashion, respectively. António et al. (2015) showed that endo-α-1,5-arabinanase and endo-β-1,4-galactanase were successful in partially degrading the arabinan and galactan side chains without destroying the backbones of the pectins [146]. Klaassen et al. (2020) significantly lowered the Gal content of RG-I type pectin by 32–67% using β-(1,4)-galactosidase, without changing the ratio of HG to RG-I [147]. Zheng et al. (2020) used endo-α-1,5-arabinanase to degrade 11.45% of the RG-I domain in pectin, mainly because of the substantial loss of Ara from the RG-I domain [148].

Different enzymes have their own unique modes of action, which allow researchers to perform targeted degradation. However, pectins with a higher degree of modification should be prepared by multi-enzyme systems rather than single enzymes. For example, Holck et al. (2011) degraded pectins gradually through multi-enzyme systems. They first separated the HG and RG-I domains of the pectins using pectin lyase and then removed the neutral sugar side chains of the RG-I domain using β-galactosidases, β-galactanase, α-arabinofuranosidase, and α-arabinanase. Finally, they prepared RG-I-type oligomers using RG-I lyase [128]. Noguchi et al. (2019) used multi-enzymes to prepare pectins with various structures; specifically, they prepared HG-type pectin by combining endo- and exo-RG-I lyase and endo-xylogalacturase. In addition, they also prepared RG-I-type pectin by endo- and exo-polygalacturonase [149]. Olawuyi et al. (2022) removed the side chains of pectin and obtained high-linearity modified products by combining polygalacturonase, galactanase, and arabinanase [150].

Enzymatic modification is influenced by the reaction temperature, pH value, substrate/enzyme concentration, and reaction time, and the first two variates are the main factors related to enzyme activities. For the reaction temperature, although there are differences between the optimal temperatures of various enzymes, most of them show the highest activity in the range of 20–60 °C. There are also a few enzymes resistant to high or low temperatures. For example, Leite et al. (2006) and Sukhumsiirchart et al. (2009) purified PMEs and PGL from guava fruit and hot springs, respectively, at the optimal temperatures of 75 °C, 85 °C, and 95 °C [137,151]. Zheng et al. (2021) cloned and heterologously expressed a cold-tolerant PGL in Escherichia coli. which could retain more than 80% of its activity at 10 °C [138]. Regarding the reaction pH value, most PME, PG, PMG, and RG-I hydrolases are more active in acidic environments, while most PAE, PGL, PMGL, and RG-I lyases are more active in alkaline environments. In addition, most neutral sugar-side-chain-degrading enzymes are maximally active in both acidic and alkaline environments (BRENDA; https://www.brenda-enzymes.org/, accessed on 7 December 2022) [152]. However, there are also some enzymes that do not follow this rule. For example, Sharma et al. (2020) and Rahman et al. (2019) prepared alkali-stable exo- and endo-PG, which showed the highest activity at pH 10.5 and 9, respectively [153,154]. Notably, in addition to the abovementioned factors, Ca^2+^ also has an important effect on the activity of PGL. Ca^2+^ can acidify the C-5 protons of GalAs to bind to the +1 subsite of PGL. Furthermore, Ca^2+^ is conducive to neutralizing the negatively charged GalAs and stabilizing the enol anion intermediate by resonance [155,156].

## 5. Potential Bioactivities of Modified Pectins

It is well-known that the structures of pectins are closely related to their bioactivities. The modification process not only provides new structures to the pectins but also changes their bioactivities. Specifically, as shown in Table 3, the modified pectins may exhibit higher or lower bioactivities than natural pectins and may also possess new bioactivities.

### 5.1. Anti-Coagulant Activity

Sulfation introduces anti-coagulant activity to modified pectins, which, generally, do not exist in natural pectins [32,41,164]. Sulfated pectins are able to prolong the thrombin time (TT), prothrombin time (PT), and the activated partial thromboplastin time (APTT), which are crucial for blood coagulation. Bae et al. (2009) even reported that these prolongation effects were stronger than those of heparin, a recognized anti-coagulant sulfated polysaccharide [30]. In addition, sulfated pectins are also confirmed to inhibit thrombin. Maas et al. (2012) and Hu et al. (2015) suggested that this inhibition effect is achieved by a mechanism dependent on antithrombin and heparin co-factor II [32,39]. However, Cipriani et al. (2009) argued that sulfated pectins can directly inhibit thrombin and factor X, being activated without this mechanism [157]. The anti-coagulant activity of sulfated pectins is proportional to their DS [31,164] and is also affected by their M_w_. Specifically, sulfated pectins with similar structures but larger M_w_ show a higher anti-coagulant activity in an in vivo experiment [157].

### 5.2. Anti-Oxidant Activity

Sulfation and selenylation enhance the anti-oxidant activity of pectins. In the former case, the sulfate groups can create a highly acidic environment in solutions, allowing them to more easily trap free radicals that can result in cell damage, such as ·OH and 2,2-diphenyl-1-picrylhydrazyl (DPPH) radicals via electrostatics [165]. In addition, sulfated pectins can also better protect cells from oxidative stress by increasing superoxide dismutase (SOD) activity and decreasing the malondialdehyde (MDA) content [12]. In the case of selenylation, selenium may exist in selenylated pectins in the form of selenyl groups or seleno-acid ester, which can form complex chelating ions, such as Fe^2+^ or Cu^2+^, thereby inhibiting the generation of ·OH [88].

In addition to modifications based on the introduction of new functional groups, those based on degradation can also improve the anti-oxidant activity of pectins. The degradation of pectin exposes or generates some functional groups at the cleavage site, such as a hydroxyl group (-OH), which can donate electrons and hydrogen, and thus are more receptive to radicals. Zhang et al. (2022), Chen et al. (2020), and Basak et al. (2022) degraded pectins by enzyme, ultrasound, and plasma treatments, and the results suggested that the modified pectins with lower M_w_ had a higher scavenging ability of ·OH, DPPH, and 2,2’-Azinobis-(3-ethylbenzthiazoline-6-sulphonate) (ABTS·+) radicals and a higher reduction ability of Fe^3+^ than that of natural pectins. A possible reason for this enhancement is that more -OH was generated during the degradation, which is effective in the scavenging of free radicals [36,81,158]. However, Wu et al. (2021) reported that as the M_w_ of modified pectin increased, the inhibition of LPS-stimulated production of ROS became stronger, but this opposite conclusion may depend not only on the increased M_w_ of modified pectin but also on the long neutral side chains attached in it [159].

However, Wu et al. (2021) reported that the inhibition effect on the generation of ROS stimulated by LPS became stronger with the increase in the M_w_, but this opposite conclusion might be drawn not only dependent on the M_w_ increase but also on the long neutral side chains attached to the modified pectin with a higher M_w_ [159]. In fact, RG-I domains with neutral side chains allow pectins to show better anti-oxidant properties, such as favorable scavenging effects on ·OH, DPPH, and ABTS·+ radicals [166].

### 5.3. Ability to Regulate the Intestinal Environment

Similar to the enhancement of the anti-oxidant activity, the introduction or removal of functional groups, such as methyl esterification and de-methyl esterification, as well as the degradation of pectins, including M_w_ and domain changes, also affect the ability of pectin to regulate the intestinal environment.

#### 5.3.1. Regulating Ability Based on Methyl Ester Groups/DM

There are several points of debate regarding the effect of methyl ester groups/DM on the ability of pectin to regulate the intestinal environment. Some researchers believe that LMPs have better potential to promote the production of short-chain fatty acids (SCFAs), such as acetic acid, propionic acid, and butyric acid [160,161,167]. However, some other researchers believe that HMPs are more capable of promoting the production of these SCFAs. For example, Larsen et al. (2019) and Fåk et al. (2015) reported that HMP could significantly boost the generation of acetic acid, propionic acid, and total SCFAs in the TIM-2 colon model, as well as the serum and caecum of obese mice, while LMP performed poorly [4,168]. In addition to the two opposite viewpoints mentioned above, there are some researchers who consider that the ability of pectins to regulate the intestinal environment is not related to the changes in the DM [169,170]. A reasonable explanation for these points of debate is that the processes of methyl esterification and de-methyl esterification are often accompanied by changes in other structural characteristics of pectins, such as M_w_, and these multi-angled changes mean that the modified pectins show different abilities to regulate the intestinal environment.

#### 5.3.2. Regulating Ability Based on M_w_

Chemical, physical, and enzymatic degradation lowers the M_w_ of pectins. Pectins with a lower M_w_ tend to possess simpler structures and lower steric resistance, meaning that they are more easily degraded by the pectinase secreted by intestinal microorganisms, thereby showing better promoting effects for the production of SCFAs and the proliferation of probiotics [171]. Chen et al. (2013) found that pectin oligosaccharides with an M_w_ less than 5 kDa increased the number of *Bifidobacteria* and *Lactobacilli* and reduced the number of *Bacteroides* and *Clostridia*. Furthermore, they also generated more acetic acid, lactic acid, and propionic acid than natural pectin [101]. Similarly, Gamonpilas et al. (2021) prepared pectin oligosaccharide with an M_w_ of 2 kDa, and it showed a better fermentation ability for the butyric-acid-producing *Eubacterium maltosivorans* strain than natural pectin with M_w_ of 200–300 kDa [17]. However, an excessively low M_w_ is unfavorable. Li et al. (2016) prepared pectin oligosaccharides with M_w_ less than 1 kDa, between 1–3 kDa, and more than 3 kDa through the multi-enzyme system. The results indicated that pectin oligosaccharide with a medium M_w_, but not the lowest M_w_ (1–3 kDa), showed the best ability to stimulate the growth of *Bifidobacterium infantis* and inhibit the growth of *Bacteroides fragilis* [163].

#### 5.3.3. Regulating Ability Based on Domains

Modification can change the proportions and structures of pectin domains to introduce them to different regulating abilities. Generally, modified pectins rich in the HG domain are likely to produce more butyric acid and acetic acid, and those containing more neutral sugar side chains tend to produce propionic acid. Cantu Jungles et al. (2019) compared the in vitro fermentation properties of pectins with or without the HG domain. The results indicated that although there was little difference in the total SCFA content between these pectins, the former could produce more acetic acid, while the latter could produce more propionic acid [172]. Similar conclusions were also drawn by Tian et al. (2016) and Ishisono et al. (2019). They both confirmed the correlation of the HG domain and neutral sugar side chains with butyric acid and propionic acid [167,173]. In addition, the types of neutral sugar in the domains with side chains also affect the regulating ability. For example, Di et al. (2017) and Onumpai et al. (2011) found that the presence of side chains rich in arabinose and galactose can result in the better proliferation of *Bifidobacteria* [170,174].

### 5.4. Anti-Tumor Activity

Pectins are broken down into smaller fragments with lower M_w_ during degradation, which are more easily absorbed by cancer cells and thus exhibit a better anti-tumor activity [175]. In addition, the simpler structures of modified pectins more easily bond to galectin-3 (Gal-3), thus preventing tumor growth [57]. Kang et al. (2006) compared the natural pectin with pectin oligosaccharides with an M_w_ less than 10 kDa, prepared by irradiation treatment, and the result suggested that the modified product significantly enhanced the inhibition of skin (B16), colon (HT29), and human melanoma (SKMEL) cancer cells [107]. Li et al. (2018) also reported that the ability of modified pectin oligosaccharides to inhibit the growth of human breast cancer cells (MCF-7) increased with the decrease in the M_w_ [57]. Not only the M_w_ but also domain change influences the anti-tumor activity of pectins. Notably, the retention and proportion of galactan side chains are crucial for the anti-tumor effect of modified pectins. Gal-3 tends to be highly expressed in tumor cells, which is closely related to the formation and metastasis of tumors [176]. A galactan side chain can occupy the binding site of Gal-3 to inhibit its activity and result in anti-tumor activity [177]. Hu et al. (2019) increased the content of Gal in natural pectin by combining the US with the NaHCO_3_-H_2_O_2_ system and found that the modified pectin had better inhibiting effects on A549 lung cancer cells than natural pectin [58]. Ellen et al. (2015) obtained modified pectins rich in HG and branched RG-I domains using acid and alkali treatments, respectively, and the latter could better inhibit the proliferation of colon cancer HT29 cells by inducing apoptosis. In addition, they reported that the arabinan and, in particular, galactan side chains were necessary for RG-I-type pectin, enabling it to show the abovementioned effects [162]. Gao et al. (2013) reported that the galactan side chain plays a key role in the ability of modified pectin to inhibit Gal-3 and is affected by the chain length. However, the arabinan side chain showed positive or negative regulation according to its position in the modified pectin [178]. Although neutral sugar side chains play a key role in the anti-tumor activity of pectins, they are not the only effective structures. Minzanova et al. (2018) suggested that even if all the neutral sugar side chains of sugar beet pectin were eliminated by alkali treatment, the remaining RG-I/HG backbones could still promote apoptosis in colon cancer cells [166].

### 5.5. Immunomodulatory Activity

Sulfation can improve the immunoregulatory activity of pectins by alleviating damage to the immune organs, stimulating the production of inflammatory cytokines, and promoting the proliferation of immune cells. Huang et al. (2020) prepared a pectin-like sulfated polysaccharide, which could relieve the weight loss and thymus index of immunosuppressed mice induced by cyclophosphamide. Furthermore, the sulfated product could better promote the release of interleukin (IL)-1β and increase concanavalin-induced T-cell proliferation, showing a higher immunomodulatory activity than natural polymer [13]. In addition, the decrease in the M_w_ also affects the immunoregulatory activity of pectins from the viewpoint of retaining the vitality of immune cells, but the specific mechanism is not well-understood [107]. The changes in the carbohydrate chain of pectins during modification also determine their bioactivity. Specifically, pectins rich in GalA, which are mainly composed of the HG domain, are able to suppress macrophage activity and inhibit the delayed-type hypersensitivity reaction. Pectins rich in branch regions, which are mainly composed of RG-I or RG-II domains, can mediate the stimulation of phagocytosis and increase the production of antibodies [166]. In addition, the different neutral sugar side chains in the branching region show immunomodulatory activity in different ways. For example, Ognyanov et al. (2013) successfully degraded the HG domain and galactan side chain through enzymatic treatment. They found that after the degradation of the HG domain, the retained RG-I domain in the modified pectin had a higher anti-complementary activity. However, this activity was significantly lowered with the removal of the galactan side chain, indicating the effect of the galactan side chain on the immunomodulatory activity of pectin [179]. Li et al. (2018) and Zhang et al. (2012) removed the arabinan side chain in pectin through acid degradation, and the former suggested that this treatment led to the greater exposure of the galactan side chain of pectin, better enabling the modified pectin to promote macrophage phagocytosis. However, the latter indicated that the removal of the arabinan side chain weakened the proliferative effect on lymphocyte and the stimulation effect on the NO secretion of the macrophage of modified pectin [48,49].

### 5.6. Anti-Inflammatory Activity

DM affects the anti-inflammatory activity of pectins. Kedir et al. (2022) suggested that LMP can reduce intestinal inflammation while HMP can diminish systemic and local inflammation. However, this mechanism is still unclear [175]. Selenylation has the potential to relieve inflammation by regulating inflammatory cytokines and oxidative stress. Tao et al. (2022) demonstrated that, compared to natural pectin, selenylation pectin provided preferable protection against ulcerative colitis by down-regulating the IL-6 and TNF-α contents and up-regulating the IL-10 content of the serum and by increasing the glutathione peroxidase (GSH-Px) activity and decreasing the myeloperoxidase (MPO) content of colon tissues [89]. In addition, Lee et al. (2018) also confirmed that selenylated pectin showed more apparent inhibition effects on the protein expression of inducible nitric oxide synthase (iNOS) in RAW264.7 cells by inhibiting the activation of p38 (related to the mitogen-activated protein kinase (MAPK) signaling pathway) [22]. The abnormal expression of iNOS is closely related to lipopolysaccharide (LPS)-induced inflammation via nitric oxide (NO) production. In addition to the MAPK signaling pathway, the expression of iNOS is also regulated by the nuclear factor-κB (NF-κB) signaling pathway. Yeung et al. (2021) indicated that compared to the natural pectin, the modified pectin with a lower M_w_ could more significantly suppress the production of nitrite and the expression of iNOS by inhibiting the LPS-induced phosphorylation of IκB kinase α/β and p65 and the degradation of IκBα. They concluded that the preferable inhibition effect of the modified pectin should be attributed to the low M_w_, which rendered it more likely to enter the cells [56]. Interestingly, the reduction in M_w_ was unfavorable regarding the anti-inflammatory activity in the research of Wu et al. (2021). They observed that the pectin oligosaccharides down-regulated the level of pro-inflammatory cytokines in an M_w_-dependent manner, and therefore, suggested that the intact pectin chain structure played an important role in the anti-inflammatory activity. They further concluded that the long neutral side chains of the intact pectins were the important structural factor [159]. Ishisono et al. (2019) also confirmed the beneficial effects of pectin side chains. Specifically, pectin with a higher content of neutral sugar side chains could better improve the damage of colonic tissue and significantly reduce the levels of IL-1β and IL-6 in the colons of colitis mice, while this effect was not observed in pectin with a lower content of neutral sugar side chains [173].

### 5.7. Hypoglycemic Activity

Although much research has been conducted on the hypoglycemic activity of natural pectins, relatively few studies have focused on the effect of modification on the hypoglycemic activity of pectins. Changes in the DM have been shown to affect this bioactivity. Chen et al. (2022) observed that the ability of modified pectins to improve insulin resistance (IR) in IR-HepG2 cells first increased and then decreased with the increase in the DM, which was in accordance with the uptake of pectins by HepG2 cells [34]. In addition, Hu et al. (2020) revealed that, compared to HMP, LMP was more easily combined with Gal-3 and showed a better protective effect on β-cells against the damage caused by oxidative stress and inflammatory stress [180].

### 5.8. Anti-Bacterial Activity

Sulfation has the ability to enhance the anti-bacterial activity of pectins, especially against Gram-negative bacteria. Bae et al. (2009) found that sulfated pectin inhibited the growth of harmful microorganisms such as *Bacillus* cereus and *Vibrio fischeri*, and the inhibitory effect on *Vibrio fischeri* at 2.0 mg/mL was approximately three times higher than that of natural pectin [30]. In addition, the decrease in the M_w_ also has an enhancing effect on the anti-bacterial activity of pectins, but it tends to inhibit the growth of Gram-positive bacteria. For example, Li et al. (2016) suggested that modified pectins with a high M_w_ prepared by enzymatic degradation had better inhibition effects on *Staphylococcus aureus*, *Bacillus subtilis*, and *Escherichia coli* than those of the modified pectin with a high M_w_ [163]. Similarly, the DM also appears to affect the effect of pectin in inhibiting Gram-positive bacteria. Jantrawut et al. (2019) combined LMP with carboxymethyl cellulose and gelatin to construct hydrogel films, which could effectively inhibit the activity of *Staphylococcus aureus* [181,182].

## 6. Conclusions and Future Prospects Perspectives

There are both advantages and disadvantages to the methods of pectin modification mentioned above. Chemical modification is the most simple and direct method that can be used to introduce new functional groups into pectin, but it requires a large amount of chemical reagents and is not environmentally friendly. Although physical modification can avoid the use of chemical reagents, showing a better capacity for environmental protection, its relatively random and uncontrollable modification mode is not conducive to preparing ideal products. Enzymatic modification is a highly targeted and efficient method, but the expensive enzymes mean that it is difficult to apply to industrial production. Based on these analyses, future research should, on the one hand, focus on the development of modification methods that combine environmental friendliness and cost-effectiveness and, on the other hand, explore methods enabling the accurate control of modification conditions that favor the targeted modification and degradation of pectins.

In addition, it is well-known that modification is able to improve the structural characteristics and promote the bioactivities of pectins and even add new bioactivities. However, it may also have a negative effect on pectins, which is ignored by most of the current research. Based on this observation, researchers should comprehensively evaluate the advantages and disadvantages of the modification of pectins and establish a multi-angle evaluation mechanism for modified pectins in future research.

Finally, although researchers have discovered a variety of changes in the bioactivity of pectins resulting from modification, there are relatively few studies that focus on the mechanisms underlying these changes. For example, some selenylated pectins have been shown to have a better capacity for the modulation of inflammatory cytokines than natural pectins, but the related signaling pathways remain unclear. Based on this observation, researchers should further explore the bioactivity mechanisms of modified pectins, especially those mechanisms involving significant changes compared to natural pectins.

## Figures and Tables

**Table 1:** Summary of the methods for chemical and physical modifications of pectins.

Modification Type	Modification Method	Mechanism/Basic Point	Main Influence Factor	Ref.
Chemical modification	Methyl esterification	Introduce -CH_3_ into the -OH on O-6 of Gal*p*A by methanol under the catalysis of acid	Acid type and concentration; reaction temperature and time	[20,28,29,30,31,32,33,34]
	De-methyl esterification	Remove -CH_3_ from Gal*p*A by alkali, acid, amidation regent, and enzyme. Alkali method: remove -CH_3_ under the catalysis of alkali to generate carboxylate and methanol; acid method: remove -CH_3_ under high temperatures and strong acid conditions; amidation treatment: ammonolyze the methyl ester groups by the ammonia in methanol in alkali conditions; enzyme method: hydrolyze the methyl ester groups by pectin methylesterase	The type and concentration of alkali, acid, amidation regent, and enzyme; reaction temperature, pH value, and time	[20,29,34,35,36,37]
	Acetylation	Introduce acetyl into the -OH on O-2 and O-3 of Gal*p*A by acetylation reagents	The type of reaction solvent and catalyst; the type and concentration of acetylation reagent; reaction temperature and time	[23,24,26]
	Sulfation	Replace the -OH of Gal*p*A with the sulfate groups by sulfation reagents	The type and concentration of sulfation reagents; reaction temperature and time	[20,31,32,33,38,39,40,41]
Chemical modification	Selenylation	Replace the -OH of Gal*p*A with selenium functional groups with inorganic selenium	The concentration of inorganic selenium; reaction temperature and time	[22,42,43]
	Acid degradation	Depolymerize pectin by using the tolerance differences between the different glycosidic bonds to acids; tolerance order: the glycosidic bonds between Gal*p*A > the glycosidic bonds between Gal*p*A and Rha*p* > the glycosidic bonds between neutral sugars	Acid type and concentration; reaction temperature and time	[44,45,46,47,48,49]
	Alkali degradation	*β*-elimination reaction: the process of cleaving C-O bonds at the *β*-position which results from the removal of the hydrogen atoms on C-5 of Gal*p*A and the formation of the double bond between C-4 and C-5	Reaction temperature, pH value, and time; cations in the reaction system	[50,51,52,53]
	Oxidative degradation	Fenton reaction: the oxidizing groups (·OH and ·O_2_^−^) generated from the decomposition of H_2_O_2_ under catalysis will combine with the hydrogen atoms attached to the carbon atoms of pectins and then attack the glycosidic bonds	Fe^2+^ concentration (for metal Fenton reaction); physical process parameters (for non-metal Fenton reaction); H_2_O_2_ concentration; reaction temperature and time	[54,55,56,57,58,59]
Physical modification	Ultrasound modification	The implosion of cavitation bubbles produced by ultrasonic power generates high shear forces, which can break the glycosidic bonds; the collapse of the cavitation bubbles promotes the dissociation of water molecules to produce -OH and -H radicals and the formation of H_2_O_2_ to attack the glycosidic bonds	US intensity/frequency; duty cycle; reaction temperature, pH value, and time	[15,16,44,58,60,61,62,63,64]
	High-pressure modification	HHP transfers pressure by liquid medium (usually water) to depolymerize pectin; HPH utilizes the forces of high-velocity impact, high-frequency vibration, cavitation, high shear stress, instantaneous pressure drop, and high pressure generated by fluid flowing through a small gap (a few hundred micrometers) in a short period of time (less than 5 s) to depolymerize pectin	Process pressure; reaction temperature, pH value, and time; cycle number (for HPH)	[65,66,67]
	Subcritical water modification	Under high temperature and pressure conditions, water is rapidly hydrolyzed to H^+^ and OH^−^, which have high catalytic activity and reactivity to depolymerize pectin	Reaction temperature and pressure; reaction pH value (acid type) and time	[68,69,70,71,72]
	Irradiation modification	Gamma irradiation and electron beam are used to modify pectin	Irradiation dosage; reaction time	[17,73,74,75,76]
	Low-temperature plasma modification	ROS, NOS, UV irradiation, electrons, and unstable radicals generated by low-temperature plasma can depolymerize pectin	Reaction voltage and time	[15,77,78,79,80,81,82]

Abbreviations: -CH_3_, methyl group; -OH, hydroxyl group; Gal*p*A, galacturonic acid residue; Rha*p*, rhamnose residue; ·OH, hydroxyl radical; ·O_2_^−^, superoxide anion radical; H_2_O_2_, hydrogen peroxide; HHP, high hydrostatic pressure; HPH, high-pressure homogenization; DHPM, dynamic high-pressure microfluidization; H^+^, hydrogen ion; OH^−^, hydroxide ions; ROS, active oxygen; NOS, active nitrogen; UV, ultraviolet.

**Table 2:** Enzymes used in enzymatic modification.

Name	Abbr.	E.C. No.	Site and Product	Ref.
Pectin methylesterase	PME	3.1.1.11	Hydrolyze methyl esters in Gal*p*A, releasing methanol.	[112]
Pectin acetylesterase	PAE	3.1.1.6	Hydrolyze acetyl esters of Gal*p*A in the HG domain, releasing ethanol.	[113]
Endo-polygalacturonase	Endo-PG	3.2.1.15	Randomly hydrolyze α-1,4-glycosidic bonds in polygalacturonic acid, releasing oligogalacturonic acid.	[114]
Exo-polygalacturonase-1	Exo-PG-1	3.2.1.67	Hydrolyze the first α-1,4-glycosidic bonds from the non-reducing end of polygalacturonic acid, releasing mono- and oligogalacturonic acid.	[115]
Exo-polygalacturonase-2	Exo-PG-2	3.2.1.82	Hydrolyze the second α-1,4-glycosidic bonds from the non-reducing end of polygalacturonic acid, releasing di- and oligogalacturonic acid.	[116]
Endo-polymethylgalacturonase	Endo-PMG	—	Randomly hydrolyze α-1,4-glycosidic bonds in methyl-esterified polygalacturonic acid, releasing methyl-esterified oligogalacturonic acid.	[117]
Exo-polymethylgalacturonase	Exo-PMG	—	Hydrolyze the first α-1,4-glycosidic bonds from the non-reducing end of the methyl-esterified polygalacturonic acid, releasing methyl-esterified mono- and oligogalacturonic acid.	[114]
Endo-polygalacturonate lyase	Endo-PGL	4.2.2.2	Randomly cleave the α-1,4-glycosidic bonds in polygalacturonic acid by a *β*-elimination reaction, releasing oligogalacturonic acid with unsaturated Gal*p*A and Gal*p*A at the terminal end, respectively.	[118]
Exo-polygalacturonate lyase	Exo-PGL	4.2.2.9	Cleave the second α-1,4-glycosidic bonds from the reducing end of polygalacturonic acid by a *β*-elimination reaction, releasing unsaturated disaccharide and shortened polygalacturonic acid.	[119]
Endo-polymethylgalacturonate lyase	Endo-PMGL	4.2.2.10	Randomly cleave the α-1,4-glycosidic bonds in methyl-esterified polygalacturonic acid by a *β*-elimination reaction, releasing oligogalacturonic acid with unsaturated methyl-esterified Gal*p*A and Gal*p*A at the terminal end, respectively.	[120]
Exo-polymethylgalacturonate lyase	Exo-PMGL	—	Cleave the first α-1,4-glycosidic bonds from the non-reducing end of methyl-esterified polygalacturonic acid by a *β*-elimination reaction, releasing unsaturated methyl-esterified mono- and oligogalacturonic acid.	[121]
Rhamnogalacturonan acetylesterase	RGAE	3.1.1.86	Hydrolyze acetyl esters of Gal*p*A in the RG-I domain, releasing ethanol.	[122]
Endo-rhamnogalacturonan hydrolase	RGH	3.2.1.171	Randomly hydrolyze the α-D-GalA-(1→2)-α-L-Rha glycosidic bonds in the RG-I backbone, releasing oligo-RG-I with Gal*p*A and Rha*p* at the reducing end and non-reducing end, respectively.	[122]
Exo-unsaturated rhamnogalacturonyl hydrolase	URGH	3.2.1.172	Hydrolyze the α-D-GalA-(1→2)-α-L-Rha glycosidic bonds of the oligo-RG-I with unsaturated Gal*p*A at the non-reducing end, releasing unsaturated glucuronic acid.	[123]
Exo-rhamnogalacturonan galacturonohydrolase	RGGH	3.2.1.173	Hydrolyze the first α-D-GalA-(1→2)-α-L-Rha glycosidic bonds from the non-reducing end in the RG-I backbone, releasing mono-GalA and oligo-RG-I.	[124]
Exo-rhamnogalacturonan rhamnohydrolase	RGRH	3.2.1.174	Hydrolyze the first α-L-Rha-(1→4)-α-D-GalA glycosidic bonds from the non-reducing end in the RG-I backbone, releasing mono-Rha and oligo-RG-I.	[125]
Endo-rhamnogalacturonan lyase	RG-I endo-lyase	4.2.2.23	Randomly cleave the α-L-Rha-(1→4)-α-D-GalA glycosidic bonds in the RG-I backbone, releasing oligo-RG-I with Rha*p* (pyranose) and unsaturated Gal*p*A (pyranose) at the reducing and non-reducing end.	[122]
Exo-rhamnogalacturonan lyase	RG-I exo-lyase	4.2.2.24	Cleave the α-L-Rha-(1→4)-α-D-GalA glycosidic bonds of the RG-I backbone with Rha*p* (pyranose) and unsaturated Gal*p*A (pyranose) at the reducing and non-reducing end, releasing disaccharide and shortened RG-I with unsaturated Gal*p*A (pyranose) at the non-reducing end.	[126]
β-D-galactosidase	—	3.2.1.23	Hydrolyze the first β-1,4-glycosidic bonds from the non-reducing end of galactan.	[122]
α-L-arabinofuranosidase	—	3.2.1.55	Hydrolyze the first α-1,5-glycosidic bonds from the non-reducing end of arabinan and AG-I.	[127]
Endo-galactanase	—	3.2.1.89	Randomly hydrolyze the β-1,4-glycosidic bonds in galactan and the β-1,3/4/6-glycosidic bonds in AG-I and AG-II.	[122]
Endo-arabinanase	—	3.2.1.99	Randomly hydrolyze the α-1,5-glycosidic bonds in arabinan and AG-I.	[127]

Abbreviations: Gal*p*A, galacturonic acid residue; Rha*p*, rhamnose residue; HG, homogalacturonan; RG-I, rhamnogalacturonan I; AG-I, type I arabinogalactan; AG-II, type II arabinogalactan.

**Table 3:** A summary of the differences between modified pectin and natural pectin.

Pectin Source	Modification Condition	Pectin Changes Caused by Modification			Ref.
		Chemical Structure and Property	Bioactivity		
Citrus	Sulfation: 1500 mg pectin + 15 mL FA + 10 mL CSA; 80–90 °C, 4 h	Sulfur content = 0→2.68%; DS = 0→0.15	Anti-coagulant activity	APTT (pectin concentration = 25–100 mg/mL): 5 times that of native pectin; longer PT	[30]
Citrus	Pectin + DMF + SO_3_-Pyr (the ratio of ηSO_3_-Pyr to -OH = 18; the ratio of total reaction volume to weight of pectin = 100); 25 °C, 6 h	DS = 0→1.41; M_w_: 4.14 × 10^5^ g/mol→1.24 × 10^5^ g/mol	Anti-coagulant activity	For each concentration increase (µg/mL) of sulfated pectin, PT and APTT increased, on average, 2.0 s and 5.7 s, respectively	[33]
*Opuntia ficus indica* cladodes	Sulfation: 400 mg pectin + 16 mL FA + 3 mL SO_3_-DMF; 50 °C, 3 h	Sulfate content = 0→2.13%, 7.04%; DS = 0→0.12, 0.46; M_w_: 7890 × 10^−3^ g/mol→3870, 2100 × 10^−3^ g/mol; Neutral sugar content: 54.20%→52.72%, 21.44%; GalA content: 31.20%→18.30%, 14.59%	Anti-coagulant activity	Longer TT and APTT	[11]
Citrus	Sulfation: 200 mg pectin + 10 mL DMSO + 4 mL SO_3_-Pyr or 200 mg pectin TBA salt + 10 mL DMSO + 6 mL SO_3_-Pyr; 80 °C, 1–3 h	Sulfate content = 0→15–25%; M_w_: 207 kDa→47–73 kDa;	Anti-coagulant activity	TT: 15.2 IU/mg; APTT: 51.96 IU/mg; inhibitory effect on thrombin (modified pectin concentration = 0.32 mg/mL): 60%	[32]
Citrus	Sulfation: 200 mg pectin + 40 mL FA + 40 mL Pyr + 16 mL CSA; 4 °C, 12 h	Almost 90% of -OH groups in pectins were sulfated	Anti-coagulant activity	Total inhibited the formation of venous thrombosis at a dose of 3.5 mg modified pectin/kg body weight of rat	[157]
*Mesona chinensis* Benth	Sulfation: 600 mg pectin + 100 mL FA + 6 mL Pyr + 3 mL CSA; 60 °C, 2 h	DS: 0→0.52; M_w_: 157 kDa→177 kDa; Uronic acid content: 29.30%→33.27%; Monosaccharide composition (molar ratio): Gal: Glu: Xyl: GalA = 0.68: 1.49: 2.54: 6.33→Gal: Glu: Xyl: GalA = 0.48: 2.43: 3.87: 6.77	Anti-oxidant activity	Scavenging abilities of DPPH radical and ·OH (pectin concentration = 1000 μg/mL): 75.11%→86.95%, 63.26%→68.93%; survival rate of RAW264.7 cells treated by H_2_O_2_ (pectin concentration = 1000 μg/mL): 78.58%→93.73%; SOD activity (pectin concentration = 1000 μg/mL): 1.12 Unit→1.29 unit; lower MDA content (pectin concentration = 1000 μg/mL): 96.88%→67.83%	[12]
*Ulmus pumila* L.	Selenylation: 1.0 g pectin + 50 mL 5% nitric acid (*v*/*v*) + sodium selenites (0.2, 0.4, 0.6, 0.8, and 1.0 g); room temperature, 24 h	Selenium content: 0→3.24, 5.14, 9.04, 10.67, 13.19 mg/g; M_w_: 2.697 × 10^9^ kDa→3.977, 4.338, 4.688, 5.220, 6.528 × 10^9^ kDa	Anti-oxidant activity	Higher reducing power; higher scavenging abilities of nitrite and ·OH; higher SOD-like scavenging activity.	[88]
Hawthorn	Degradation by enzymes: 1% pectin solution (*w*/*v*) + enzymes, 40–50 °C, pH 3.5–7.5, 4–7 h	DM: 55.73%→38.56%, 41.05%, 26.62%; 40.46%; GalA content: 88.84%→82.82%, 92.01%, 69.47%; 90.04%; M_w_: 288.41 kDa→6.51, 37.18, 3.59, 7.01 kDa	Anti-oxidant activity	IC 50 (·OH scavenging activity): 1.08 mg/mL→0.335, 0.779, 0.717, 0.481 mg/mL; IC 50 (ABTS·+ radical scavenging activity): 2.06 mg/mL→1.807, 1.924, 1.272, 1.874 mg/mL	[36]
Raspberry	Degradation by ultrasound: 2 mg/mL pectin + 50 mL distilled water; sonication, ice-bath, 400 W, 60 min	DM = 31.49%→28.07%; Neutral sugar = 21.42%→18.03%; M_w_ = 15.02 kDa→11.01 kDa; monosaccharide composition (%) = Gal: Rha: Ara: Glc: Xyl: Man: GalA = 8.45: 2.39: 9.15: 13.65: 2.17: 1.65: 62.48→8.14: 1.79: 7.08: 12.03: 1.62: 1.49: 67.76	Anti-oxidant activity	DPPH radical scavenging activity: 0.75 mg AAE/mg→0.94 mg AAE/mg; ABTS·+ radical scavenging activity: 0.18 mg AAE/mg→0.32 mg AAE/mg; ferric reducing power: 0.39 mg AAE/mg→0.44 mg AAE/mg; The activity of human hepatic L02 cells with pre-treatment by pectins after H_2_O_2_ treatment (pectin concentration = 0.1 mg/mL): 83.39%→92.35%	[158]
Citrus	Degradation by irradiation: 2% pectin solution (*w*/*v*); irradiated (20 kGy, 14 °C,10 kGy/h)	M_w_: 500 kDa→37 kDa	Anti-oxidant activity	β-carotene retention in β-carotene-linoleic acid bleaching assay (pectin concentration = 20 mg/mL): 21%→59%; DPPH radical scavenging activity (pectin concentration = 4 mg/mL): less than 20%→60%	[107]
Apple	Degradation by plasma: 10 g pectin + plasma treatment (230 V,1.2 kHz, 3, 6, 9, 12, 15 min);	M_w_: 160,677.4 g/mol→about 40,000–12,000 g/mol; DE: 68.12%→54.21–65.78%; GalA content: 0.653%→0.658–0.816%	Anti-oxidant activity	Fe^3+^ reducing power (pectin concentration = 0.5% and 2%, plasma treatment = 15 min): 2.67 and 1.85 times that of natural pectin; higher DPPH radical scavenging activity	[81]
Citrus canning processing water	Degradation by ultrasound: pectin + 50 mM H_2_O_2_ & 10 mM ascorbic acid (0.5% *m*/*v*); ultrasound (3.8 W/mL, 22 kHz), 30 °C, 60 min	M_w_ of modified pectin: 2113, 3683, 7469 g/mol; Monosaccharide composition (molar ratio) of modified pectin: Ara: Gal: Rha: GalA: Glc: Xyl = 39.30: 7.85: 2.19: 45.15: 4.1: 1.59 (lowest M_w_); 44.3: 7.79: 3.49: 42.31: 0.85: 0.78 (medium M_w_); 50.47: 13.44: 11.23: 20.68: 2.8 (highest M_w_)	Anti-oxidant activity	Lower inhibitory effect on the generation of ROS with the decrease in M_w_	[159]
Citrus	Degradation by acid, alkali, enzymes, acid + enzymes, and alkali + enzymes	M_w_ of modified pectin: 1.39, 0.51, 0.95, 1.41, 1.12 × 10^5^ g/mol; DM of modified pectin: 71.41%, 2.02%, 68.80%, 64.70%; 14.81%	Ability to regulate the intestinal environment	Modified pectin with M_w_ of 1.12 × 10^5^ g/mol and DM of 14.81% is best for producing acetic acid, propionic acid, and butyric acid	[160]
—	Methyl esterification: 100 g pectin + 800 mL methanol/40 mL concentrated H_2_SO_4_, 6 d; then 1 L methanol/37.5 mL concentrated H_2_SO_4_, 4 °C, 6 d	DM of modified pectin: 34.4%, 70.8%, 92.6%; GalA content of modified pectin: 74.4%, 65.5%, 77.9%	Ability to regulate the intestinal environment	Acetate, propionate, butyrate, and total SCFAs content (pectin DM 34.4%→92.6%): 86.25%→71.62%; 15.11%→11.05%; 9.08%→5.77%; 110.62%→88.71%; *Lactobacillus* counts (21 d feeding, pectin DM 34.4%→92.6%): 7.60 log→6.26 log	[161]
Orange, lemon, lime, and sugar beet	De-methyl esterification and amidation	DM of modified pectin: 11.4–74.7%; Gal content of modified pectin: 9.1–30.7%; Ara content of modified pectin: 0.8–16.7%; Rha content of modified pectin: 1.7–3.7%; Glc content of modified pectin: 0.7–8.7%; GalA content of modified pectin: 70.7–83.0%	Ability to regulate the intestinal environment	Higher stimulation of *F. prausnitzii* with the increase in DM	[4]
Apple	Degradation by high pressure: 1.84% pectin solution (*w*/*v*); 155 MPa, 63 °C, cycle number = 6 passes	Monosaccharide composition (wt%): GalA: Ara: Gal: Rha: Glc: Xyl = 71.68: 6.61: 7.45: 3.48: 0.56: 2.91→29.56: 18.35: 21.12: 9.12: 1.52: 8.42	Ability to regulate the intestinal environment	Fermentation in vivo for 24 h: *Bacteroides* number: 8.54 log→7.85 log; *Clostridia* number: 7.91 log→7.21 log; total SCFAs content: 39.74 mM→59.85 mM; acetic acid content: 23.92 mM→31.64 mM; lactic acid content: 2.70 mM→12.57 mM; propionic acid content: 5.17 mM→7.89 mM	[101]
Pomelo	Degradation by irradiation: 40 mL 1% pectin solution (*w*/*v*), 3–250 kGy	DM: 68.2%→64.4% (40 kGy) and 72.4% (125 kGy); M_w_: 193129 Da→9428 Da (40 kGy) and 1835 Da (125 kGy); monosaccharide composition (molar ratio): Fuc: Rha: Ara: Gal: Glc: Xyl: Man: GalA = 0.18: 3.52: 11.64: 9.36: 1.84: 1.18: 1.12: 71.15→0.21: 3.40: 10.46: 7.27: 1.95: 1.26: 1.49: 73.96 (40 kGy) and 0.22: 1.66: 10.56: 3.70: 2.13: 1.08: 1.28: 79.37 (125 kGy)	Ability to regulate the intestinal environment	Produce more butyric acid; increase the number of *Eubacterium maltosivorans* strain	[17]
Citrus	Degradation by irradiation: 2% pectin solution (*w*/*v*); irradiated (20 kGy, 14 °C,10 kGy/h)	M_w_: 500 kDa→37 kDa	Anti-tumor activity	Higher inhibitory effects on skin (B16), colon (HT29), and human melanoma (SKMEL) cancer cells	[107]
Citrus	Degradation by ultrasound: pectin + 50 mM H_2_O_2_ & 10 mM ascorbic acid (0.5% *m*/*v*); ultrasound (11.4 W/mL, 22 kHz), 50 °C, 60 min	M_w_: 791 kDa→7.65 kDa; monosaccharide composition (molar ratio): Ara: GalA: Gal: Rha: Fuc: Xyl = 44.55: 22.3: 18.4: 11.49: 2.3: 0.91→48.2: 14.36: 18.58: 13.46: 4.22: 1.18	Anti-tumor activity	Cell viability of human breast cancer cell MCF-7 (pectin concentration = 500 µg/mL): 34.71%→56.39%; LDH content (pectin concentration = 500 µg/mL): 162.8% compared to the control group→138.3% compared to the control group	[57]
Citrus	Degradation by ultrasound: 25 mL pectin solution (5 mg/mL) + M_H2O2_: M_NaHCO3_ = 1: 2.5, 11.4 W/mL, 50 °C, 50 min	M_w_: 1088 kDa→33 kDa; monosaccharide composition (molar ratio): Man: Rha: GlcA: GalA: Glc: Gal: Ara: Fuc = 1.12: 3.98: 0.49: 59.07: 4.84: 12.67: 15.94: 0.27→1.2: 4.81: 0.72: 54.39: 3.62: 16.42: 17.05: 1.81	Anti-tumor activity	Growth inhibition rate of A549 lung cancer cell (pectin concentration = 1 mg/mL): about 15%→27.21%	[58]
Sugar beet	Degradation by enzymes: β-galactosidase + endo-galactanase (without galactan side chain); α-L-arabinofuranosidase + endo-arabinose + β-galactosidase + endo-galactanase (without all side chains)	β-1,4-galactan content: 100%→4–13% (without galactan side chain), 4–20% (without all side chains); terminal Ara content: 100%→36% (without galactan side chain), 36% (without all side chains)	Anti-tumor activity	Lower inhibitory effect on the proliferation of colon cancer cell HT29 as the removal of the galactan side chain	[162]
Chinese yam	Sulfation: 400 mg pectin + 100 mL FA + 10 mL CSA & Pyr (1: 3, *v*/*v*); 70 °C, 3 h	DS: 0→0.51; M_w_: 33.33 kDa→37.04 kDa; Uronic acid: 21.15%→27.55%; monosaccharide composition (molar ratio): Rha:Gal:Glc:Xyl:GalA:GlcA = 1.77:11.36:13.44:1.53:15.47:1.67→0.13:13.51:12.98:1.25:16.22:0.91	Immunomodulatory activity	Higher thymus index in immunosuppressed mice; higher proliferation effect on concanavalin-induced T-cells in high dosage; the absolute number of CD3^+^CD4^+^ (pectin concentration = 200 mg/kg body weight): 52.07→55.87; the ratio of CD4^+^/CD8^+^ (pectin concentration = 200 mg/kg body weight): 3.50→3.67; higher contents of TNF-α and IL-1β in high dosage; lower levels of serum antibody in high dosage	[13]
Citrus	Degradation by irradiation: 2% pectin solution (*w*/*v*); irradiated (20 kGy, 14 °C,10 kGy/h)	M_w_: 500 kDa→37 kDa	Immunomodulatory activity	Spleen cell viability (pectin concentration = 1 mg/mL): 33.3%→69.3%	[107]
Bee pollen of *Nelumbo nucifera*	Degradation by acid: pectin + 0.1 M TFA, 80 °C, 8 h	M_w_: 380 kDa→9 kDa; monosaccharide composition (molar ratio): Rha:GalA:Gal:Ara:GlcA:Glc = 11.5:12.0:41.2:29.7:2.0:3.6→15.3:18.1:50.7:0:13.3:2.7	Immunomodulatory activity	Higher stimulative effect on macrophage phagocytosis; lower NO content	[48]
*Panax ginseng*	Degradation by acid: pectin + 0.1 M TFA, 80 °C, 6 h	M_w_: 110 kDa→40 kDa; monosaccharide composition (molar ratio): Ara:Gal:Glc:Rha:GalA:GlcA = 40.9:44.4:2.9:4.1:5.3:2.0→0:76.1:0.6:10.1:8.6:4.6	Immunomodulatory activity	Lower stimulative effect on lymphocyte proliferation; lower NO content	[49]
Apple	Selenylation: 1 g pectin + 100 mL 0.5% nitric acid solution (*v*/*v*) + 1 g sodium selenite; ultrasound treatment 240 W, 75 °C, 6 h	M_w_: 517 kDa + 64.9 kDa + 18.8 kDa→603 kDa; monosaccharide composition (molar ratio): Rha:Ara:Gal:Glc:Xyl:GalA = 0.004:0.017:0.256:0.080:0.111:0.492→0.032:0.005:0.225:0.067:0.072:0.599	Anti-inflammatory activity	The reduction in colon length (pectin concentration = 200 mg/mL): 13%→7%; lower IL-6 and TNF-α content; higher IL-10 content; higher activity of glutathione peroxidase; lower MPO content	[89]
*Ulmus Pumila* L.	Selenylation: 1 g pectin + 50 mL 0.5% nitric acid solution (*v*/*v*) + 0.2 g and 0.4 g sodium selenite, room temperature, 24 h	—	Anti-inflammatory activity	Higher inhibiting effects on the activation of p38 and the protein expression of iNOS; lower NO content	[22]
Okra pod	Degradation by Fenton reaction: 500 mL 1% pectin solution (*v*/*v*) deionized water + 3 mM, 5 mM, and 7 mM granular FeSO_4_ + 500 mL 2% H_2_O_2_ (*v*/*v*), room temperature, 2 h	M_w_: 112.31 + 2.22 + 1.15 + 0.53 kDa→6.09 + 1.02 kDa (3 mM FeSO_4_), 4.89 + 2.48 + 1.22 kDa (5 mM FeSO_4_), 3.97 + 1.79 + 0.87 kDa (7 mM FeSO_4_); GalA content: 28.23 wt%→22.39, 13.61, 0.58 wt%; monosaccharide composition (molar ratio): Rha:Ara:Xyl:Man:Glu:Gal = 37.73:8.23:2.40:1.38:3.04:47.23→62.29:2.32:2.53:6.03:3.62:23.22 (3 mM FeSO_4_), 72.05:1.58:2.42:6.52:4.12:13.30 (5 mM FeSO_4_), 82.99:0.42:0.70:7.43:4.54:3.92 (7 mM FeSO_4_)	Anti-inflammatory activity	Higher inhibiting effects on the production of nitrite, the expression of iNOS, the phosphorylation of IκB kinase α/β and p65, and the degradation of IκBα	[56]
*Ficus pumila* L.	Methyl esterification: acidified methanol: 48.22 mL anhydrous MeOH + 1.78 mL acetyl chloride, 25 °C, 1 h; 200 mg pectin + 20 mL acidified methanol, 0.5–14 h; De-methyl esterification: 200 mg pectin + 0.02 M and 0.03 M NaOH, 4 °C, 30 min	DM of modified pectins: 3%, 16%, 25%, 34%, 45%, 54%, 63%, 76%, 84%, 94%; M_w_ of modified pectins: 42.7, 46.5, 50.5, 51.5, 52.6, 67.3, 48.4, 45.7, 43.8, 38.0 kDa	Hypoglycemic activity	Glucose consumption in IR-HepG2 cells: when DM is between 3% and 54%: DM3 < DM 16 < DM 25 < DM 34 < DM 45 < DM54; when DM is between 54% and 94%: DM54 > DM 63 > DM 76 > DM 84 > DM 94	[21]
Citrus	Sulfation: 1.5 g pectin + 15 mL FA + 10 mL CSA, 80–90 °C, 4 h	Sulfur content = 0→2.68%; DS = 0→0.15	Anti-bacterial activity	Inhibitory effect on *Bacillus cereus* and *Vibrio fischeri* (pectin concentration = 2.0 mg/mL): about 3%→20%, about 5%→58%	[30]
Orange peel	Degradation by enzymes: 4% pectin solution (*w*/*v*) + crude enzyme (pectinase (48.3 U/mL) + filter paper cellulase (FPase, 0.2 U/mL) + CMCase (2.8 U/mL) + xylanase (50.0 U/mL)), 45 °C, 6 h; then filter (membrane cut-off of 3 kDa and 1 kDa)	DPp of modified pectins: 6, 9, 19; monosaccharide composition (%): Rha:Ara:Gal:Glc:Xyl:Fru:GalA = 7.8:13.7:5.0:30.3:19.4:2.1:21.7→2.5:32.6:8.8:34.8:6.9:1.3:13.2 (modified pectin with DPp of 6), 3.2:17.6:14.8:36.9:1.3:2.0:24.2 (modified pectin with DPp of 9), 6.2:22.0:12.3:39.7:2.0:1.4:16.4 (modified pectin with DPp of 19)	Anti-bacterial activity	Minimum inhibitory concentrations of modified pectins against *Staphylococcus aureus*, *Bacillus subtilis*, and *Escherichia coli*. (mg/mL): 12.5, 12.5, 25 (modified pectin with DPp of 6); 12.5, 12.5, 25 (modified pectin with DPp of 9); 25, 25, 50 (modified pectin with DPp of 19)	[163]

Abbreviation: M_w_, molecular weight; DS, the degree of sulfation; DM, the degree of methyl esterification; Gal, galactose; Ara, arabinose; GalA: galacturonic acid; Rha, rhamnose; Man, mannose; Xyl, xylose; Glc, glucose; Fru, fructose; Fuc, fucose; GlcA, glucuronic acid; CSA, chlorosulfonic acid; DMF, *N*, *N*-dimethylformamide; Pyr, pyridine; FA, formamide; DMSO, dimethyl sulfoxide; TBA, thiobarbituric acid; PT, prothrombin time; APTT, activated partial thromboplastin time; TT, thrombin time; LDH, lactate dehydrogenase; AAE, ascorbic acid equivalent; DPPH, 2,2-Diphenyl-1-picrylhydrazyl; SOD, superoxide dismutase; MDA, malondialdehyde; ABTS, 2,2’-Azinobis-(3-ethylbenzthiazoline-6-sulphonate); ROS, reactive oxygen species; SCFA, short-chain fatty acid; TNF-α, tumor necrosis factor-α; IL-1β, interleukin-1β; NO, nitric oxide; MPO, nitric oxide; iNOS, inducible nitric oxide synthase; IR, insulin resistance; DPp, the average degree of polymerization.

## Data Availability

No new data were created or analyzed in this study. Data sharing is not applicable to this article.
